# A Comparative Evaluation of Surgical Techniques Applied in Lumbar Synovial Cysts in Accordance with the Literature

**DOI:** 10.3390/diagnostics15141767

**Published:** 2025-07-13

**Authors:** Mustafa Emre Sarac, Zeki Boga

**Affiliations:** Department of Neurosurgery, Adana City Training and Research Hospital, 01370 Adana, Turkey; zekiboga2013@gmail.com

**Keywords:** spinal fusion, joint instability, synovial cyst, comparative study

## Abstract

**Background/Objectives:** The aim of this study was to compare the clinical outcomes of cyst excision alone and the combination of excision and unilateral dynamic instrumentation in the surgical treatment of lumbar synovial cysts at the L4–L5 level and to determine which surgical approach is more effective. **Methods:** Thirty-three patients who underwent operations on the L4–L5-level synovial cyst in a single center between January 2015 and January 2021 were included in this retrospective study. Patients were divided into two groups: cyst excision only (*n* = 18) and excision with unilateral dynamic instrumentation (*n* = 15). The pain levels of the patients were assessed by VAS score, and their functional status was assessed by the Oswestry Disability Index. The mean follow-up period was 28.2 ± 4.0 months in the excision group and 27.6 ± 4.4 months in the instrumentation group. **Results:** VAS and ODI scores improved significantly in both groups (*p* < 0.001). The improvement in low back pain VAS scores was more significant in the instrumentation group (delta VAS: −5.8 ± 1.3 vs. −5.0 ± 1.2, *p* = 0.042). The complication rate was 27.8% in the excision group and 13.3% in the instrumentation group. The development of listhesis was significantly more frequent in the excision group (16.7% vs. 0%, *p* = 0.028). Reoperation rates were 16.7% in the excision group and 6.7% in the instrumentation group. **Conclusion:** Although both surgical approaches are effective in the treatment of synovial cysts at the L4–L5 level, the addition of dynamic instrumentation unilaterally seems to be more advantageous, especially in the control of low back pain and prevention of listhesis risk. The choice of surgical technique should be individualized by considering patient-specific factors.

## 1. Introduction

Lumbar facet cysts have become an increasingly common clinical problem in recent years, with advances in imaging technologies facilitating their diagnosis [1]. These lesions, also termed juxtafacet cysts in the literature, encompass both synovial and ganglion cysts. Despite their histopathological differences, these cysts present similar clinical features and treatment outcomes and likely represent various manifestations of the same degenerative process [2,3]. Research in the field of biomechanics demonstrates that L4–L5 synovial cysts show distinct stress distribution compared to other spinal segments, which indicates the need for level-specific treatment methods [4,5,6].

Lumbar synovial cysts occur most frequently at the L4–L5 level and constitute a significant cause of radiculopathy and back pain. The optimal management approach for lumbar synovial cysts remains debated. While conservative treatments are typically recommended initially, long-term success rates reported in the literature have been suboptimal. Though surgical intervention has demonstrated efficacy in numerous studies, there is no established consensus regarding the necessity of adding instrumentation [7,8,9]. Considerable controversy persists regarding their optimal surgical management. While some research indicates favorable outcomes with simple cyst excision, others support more comprehensive approaches, including concomitant instrumentation, particularly in cases presenting with underlying instability or spondylolisthesis [10]. Additionally, factors influencing postoperative outcomes and recurrence rates are not well established in the current literature [11,12]. Uncertainties in surgical treatment options highlight the importance of developing new surgical methods and establishing long-term follow-up strategies. This research introduces unilateral polyetheretherketone (PEEK) rod instrumentation as a new approach because finite element analysis models show that this material produces 30% less stress concentration at adjacent segments than traditional titanium rods [4,6].

The previous research on this rare pathology has been restricted by small sample sizes and the inclusion of multiple spinal levels which made the findings impossible to compare. Ganga et al. [8] performed a comprehensive meta-analysis of 1251 patients across multiple lumbar levels and various surgical techniques, but their study involved patients with different biomechanical characteristics at each spinal level. Chen et al. [13] conducted a meta-analysis of minimally invasive tubular approaches but involved patients from L1-S1 levels which have different mechanical properties and surgical considerations. The need exists for studies that evaluate different surgical techniques at the same spinal level with assessment of long-term results [8]. Research focused on the L4–L5 level would produce more consistent results because this level is most commonly affected and its biomechanical properties are well characterized. This study provides the first direct assessment of surgical outcomes at the L4–L5 level among patients with uniform characteristics to address the main issue found in previous multi-level research.

The current method introduces main innovations through its exclusive focus on the L4–L5 level to reduce inter-level biomechanical variability and through its use of unilateral PEEK rod dynamic stabilization systems with documented elastic modulus compatibility to bone tissue in light of standardized radiological criteria such as dynamic flexion-extension analysis for objective assessment.

The current study’s objective was to evaluate the efficacy of two surgical approaches (cyst excision only versus excision with unilateral instrumentation) in treating lumbar synovial cysts at the L4–L5 level, based on both clinical and radiological outcomes. The instrumentation systems employed offer advantages regarding bone elastic modulus compatibility and help maintain segmental movement, thereby preventing iatrogenic instability. Previous studies have demonstrated that these systems reduce stress concentrations at the bone–screw interface and adjacent segments [4,6]. This study addresses methodological limitations of previous research through detailed biomechanical considerations and standardized radiological protocols to provide evidence-based surgical decision-making guidance. This evaluation seeks to determine the effectiveness of specific surgical techniques, providing clearer guidance for treatment selection. To test our hypothesis, we compared outcomes between these two surgical approaches in patients with lumbar synovial cysts.

## 2. Method

### 2.1. Study Design and Patient Selection

All patients diagnosed with lumbar synovial cysts who underwent surgical treatment between January 2015 and January 2021 were screened retrospectively, and 33 patients with primary synovialcysts associated with degenerative processes at the L4–L5 level were included. A priori power analysis was performed using G*Power 3.1.9.7 based on previous studies reporting VAS score differences. With an effect size of 0.8, alpha of 0.05, and power of 0.80, the required sample size was calculated as 26 patients (13 per group). Our sample size of 33 patients exceeded this requirement.

**Figure 1:**
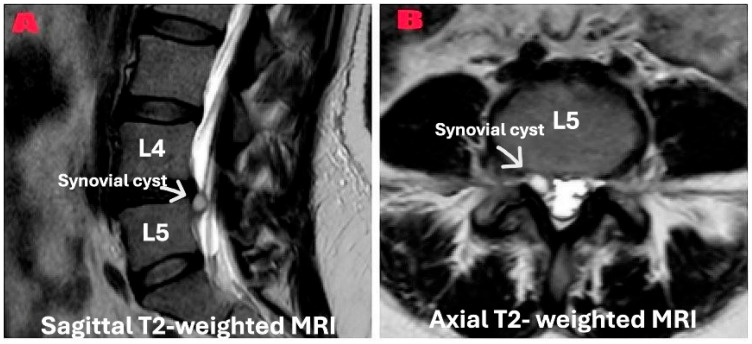
Preoperative T2-weighted magnetic resonance imaging demonstrating lumbar synovial cyst at the L4–L5 level. (**A**) A sagittal T2-weighted image showing the synovial cyst (indicated by the white arrow) as a well-defined, fluid-intensity lesion in the posterior spinal canal between the L4 and L5 vertebrae, causing the compression of the dural sac. (**B**) An axial T2-weighted image of the same patient revealing the synovial cyst (indicated by the white arrow) in the right posterolateral aspect of the spinal canal, demonstrating a significant mass effect on the thecal sac and neural elements. The cyst exhibits high signal intensity in T2-weighted sequences because of its fluid content.

**Figure 2:**
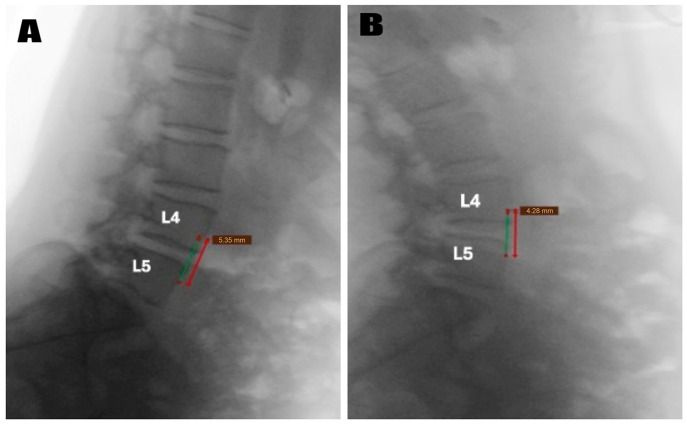
Preoperative dynamic lateral radiographs of the same patient, demonstrating segmental instability at the L4–L5 level. (**A**) A flexion radiograph showing 5.35 mm anterior translation of L4 over L5 as measured from the vertebral body reference lines (red and green lines). (**B**) An extension radiograph showing 4.28 mm anterior displacement of L4 over L5 as measured from the vertebral body reference lines. The measurements demonstrate Grade 1 spondylolisthesis according to Meyerding classification. Both images confirm segmental instability that warrants unilateral dynamic stabilization in addition to cyst excision.

**Figure 3:**
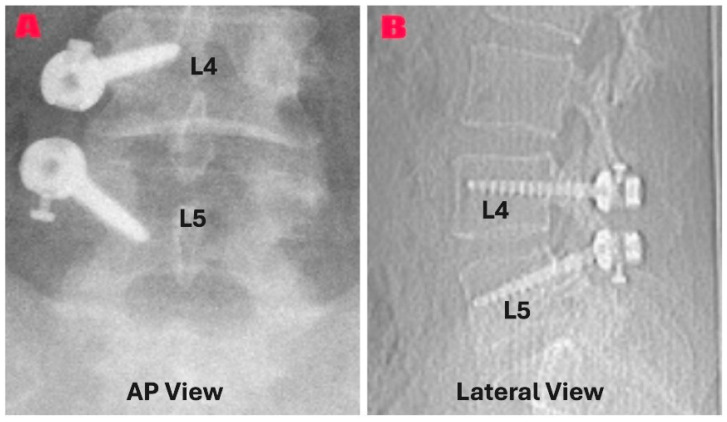
Postoperative imaging that shows unilateral PEEK rod instrumentation at the L4–L5 level following a synovial cyst excision. (**A**) An anteroposterior radiograph showing the proper placement of unilateral pedicle screws in the L4 and L5 vertebrae (**B**). The CT scan showing the PEEK rod and the pedicle screws applied unilaterally and properly reveals that the segmental alignment has been maintained.

Preoperative MRI confirmed the diagnosis radiologically (Figure 1). Dynamic flexion-extension radiographs were obtained in all patients preoperatively (Figure 2) to evaluate segmental instability and to minimize selection bias that may occur due to the retrospective nature of the study. Cyst excision alone was performed in patients without instability according to imaging results, and unilateral dynamic instrumentation was performed (Figure 3) in addition to excision in patients with grade 1 instability according to the Meyerding classification. The systematic protocol used for decision-making reduced the impact of personal choices on selection of treatment. Patients with spondylolisthesis above grade 1 were not involved in the study. Exclusion of these patients provided sample homogeneity in addition to the biomechanic success of PEEK rod systems in mildly unstable cases due to their flexible designs.

### 2.2. Inclusion and Exclusion Criteria

Inclusion criteria: (1) Age over 18 years; (2) histopathologically confirmed synovial cyst at the L4–L5 level; (3) minimum 2-year regular follow-up; and (4) confirmed diagnosis by preoperative imaging.

Exclusion criteria: (1) Previous lumbar surgery; (2) history of spinal trauma, infection, and inflammatory diseases; (3) history of primary or metastatic tumors; (4) patients with more than grade 1 translation, according to the Meyerding classification, on dynamic radiographs.

### 2.3. Radiological Assessment and Instability Grading

The radiological evaluation for all patients included the following: In addition to preoperative lumbar MRI and early postoperative lumbar CT in all patients, dynamic flexion–extension radiographs were obtained to evaluate instability preoperatively and postoperatively. Meyerding classification was used in the evaluation of listhesis.

### 2.4. PEEK Rod System Specifications

The unilateral instrumentation utilized PEEK-OPTIMA™ Natural LT1 rods (Invibio Ltd., Thornton-Cleveleys, UK) with specific mechanical properties that were optimized for dynamic stabilization. The system employed 40–50 mm length rods, depending on patient anatomy at the L4–L5 level. The mechanical properties of this material include an elastic modulus of 3.6–4.1 GPa (compared to 110 GPa for titanium), tensile strength of 179 MPa, flexural strength of 179 MPa, and density of 1.30 g/cm^3^. As demonstrated in finite element analysis studies, these properties provide a reduction of approximately 30% in stress concentration at adjacent segments, compared to traditional titanium rods [4,6]. The PEEK material’s elastic modulus more closely matches that of cortical bone (18 GPa), thereby reducing stress shielding effects and preserving physiological load transmission.

### 2.5. Surgical Technique and Patient Groups

All surgeries were performed under general anesthesia in the prone position on a radiolucent table. After sterile preparation, the L4–L5 level was identified by fluoroscopy. A midline incision was made, and paravertebral muscles were dissected subperiosteally.

In the excision-only group (*n* = 18), the facet joint and lamina were exposed microscopically. The cyst was accessed through minimal laminotomy and/or partial facetectomy. The cyst wall was carefully dissected from neural structures and completely separated from surrounding tissues. Total excision was achieved, and tissue was sent for pathological examination. Foraminotomy was performed when necessary to decompress nerve roots. After hemostasis, tissues were closed anatomically.

In the group composed of patients who underwent excision with unilateral instrumentation (*n* = 15), wider exposure was provided on the cyst side. Following the pedicle screws were placed and verified by fluoroscopy, cyst excision was performed using the same technique. After hemostasis, pedicle screws were connected with a PEEK rod. Facet joint mobility was assessed before and after instrumentation. Closure was performed in anatomical layers. Figure 3 shows a representative postoperative radiograph (A) and CT scan (B) from the instrumentation group, demonstrating pedicle screw placement in anteroposterior (A) and lateral (B) views.

Postoperatively, mobilization began on day one in both groups. Patients wore lumbar corsets for 3 weeks in the excision group and for 6 weeks in the instrumented group. Physiotherapy started at week 6, with a return to normal activities planned at week 6 and week 12 for the respective groups. Restrictions on heavy activities were evaluated at the 6-month follow-up.

All patients received prophylactic antibiotics, microscopic visualization to prevent dural injury, and regular neurological examinations. The first follow-up occurred at one week, with suture removal.

Our sample included all eligible L4–L5-level cyst patients operated on during the study period. Post hoc power analysis using G*Power 3.1.9.7 showed 62% power for VAS score changes with our sample size (*n* = 33), consistent with the literature for this rare pathology.

### 2.6. Clinical Evaluation and Follow-Up

Patient evaluation employed validated scales: the Visual Analogue Scale (VAS) for back and leg pain and the Oswestry Disability Index (ODI) for functional assessment. These were measured preoperatively, early postoperatively, and during follow-ups. Follow-up visits occurred at 1, 3, 6, 12, and 24 months postoperatively, including neurological examination and evaluation of pain, function, complication, and radiological instability.

### 2.7. Statistical Analysis

Data were analyzed using SPSS 23.0. Kolmogorov–Smirnov testing was used to assess normality. Chi-square and Fisher’s exact tests were used to compare categorical variables. Independent *t*-tests compared groups, while paired *t*-tests analyzed intra-group changes. This study used multivariate logistic regression to determine which factors independently predicted complications and reoperation among age, gender, preoperative instability grade, and surgical technique. Propensity score matching was considered but not performed due to sample size limitations; this was acknowledged as a study limitation. Cohen’s d was used to calculate effect sizes, classified as negligible (<0.20), small (0.20–0.49), moderate (0.50–0.79), and large (≥0.80). Significance was set at *p* < 0.05.

### 2.8. Complication Evaluation

Complications were monitored for a minimum duration of one month. Early complications (wound problems, infection) and late complications (instability, implant issues, recurrence) were documented separately. Treatment strategies, outcomes, and reoperation cases were analyzed in detail.

### 2.9. Ethical Approval and Informed Consent

Approval was obtained from the Ethics Committee of Health Sciences University, Adana City Training and Research Hospital (Decision no: 2025/336, Meeting number: 10, Date: 6 February 2025) Adana, Türkiye. In accordance with the hospital’s standard protocol, all patients had provided written informed consent prior to surgery, which included explicit permission for the use of anonymized clinical data for scientific and academic purposes. Therefore, no additional informed consent specific to this study was required or obtained.

## 3. Results

The primary outcome of this study is pain improvement as measured by changes in VAS score for back and leg pain. Secondary outcomes include functional improvement (ODI scores), complication rates, reoperation rates, and surgical parameters (operation time, hospital stay). While our a priori power analysis demonstrated 80% power for primary outcomes, post hoc analysis revealed 62% power for certain secondary outcomes, which is acceptable for this rare pathology and provides meaningful clinical insights despite being below the conventional 80% threshold.

A total of 33 patients with synovial cysts at the L4–L5 level were included in our study. Of these, 18 patients underwent cyst excision only, and 15 patients underwent additional instrumentation. The mean age was 50.8 ± 9.6 years in the excision-only group and 47.9 ± 10.2 years in the instrumentation group. Female patients predominated in both groups: there were 11 females (61.1%) and 7 males (38.9%) in the excision group, and 9 females (60.0%) and 6 males (40.0%) in the instrumentation group (Table 1).

The mean duration of symptoms was 8.1 ± 4.0 months in the excision group and 7.6 ± 3.8 months in the instrumentation group. The most prevalent presenting symptom was radicular leg pain (94.4% in the excision group, 93.3% in the instrumentation group), followed by axial low back pain (88.9% in the excision group, 86.7% in the instrumentation group) and neurogenic claudication (61.1% in the excision group, 60.0% in the instrumentation group). No statistically significant differences were found between groups for these demographic and clinical characteristics (Table 1).

**Figure 4:**
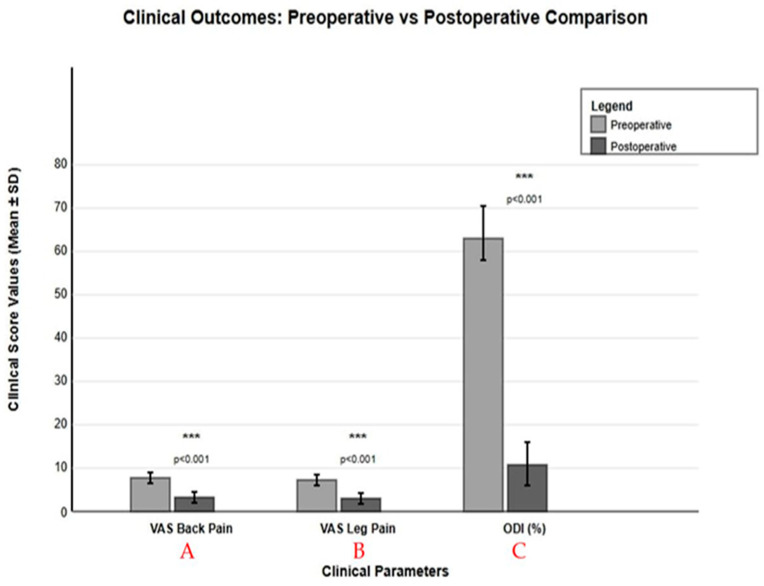
Comparison between clinical outcomes of preoperative and postoperative assessments. Significant improvements were observed in all clinical parameters following surgical intervention. (**A**) Visual Analogue Scale scores for back pain decreased from 7.6 ± 1.2 to 2.2 ± 1.1 (*p* < 0.001). (**B**) Visual Analogue Scale scores for leg pain decreased from 7.4 ± 1.25 to 2.1 ± 1.05 (*p* < 0.001). (**C**) Oswestry Disability Index scores decreased from 62.9 ± 12.2% to 21.3 ± 8.5% (*p* < 0.001). Data presented as mean ± standard deviation with error bars representing standard deviation. Statistical significance was determined using paired *t*-test with *** *p* < 0.001, and data was combined from both surgical groups (*n* = 33 patients, mean follow-up 28 months).

Both surgical approaches demonstrated significant clinical improvements. In the excision group, VASback pain decreased by 5.0 ± 1.2 points, and VASleg pain decreased by 5.5 ± 1.3 points (both *p* < 0.001). The instrumentation group showed VASback pain reduction of 5.8 ± 1.3 points and VASleg pain reduction of 5.1 ± 1.2 points (both *p* < 0.001). Functional improvement measured by ODI was 42.2 ± 11.2% in the excision group and 41.0 ± 11.8% in the instrumentation group. The instrumentation group demonstrated superior low back pain control (delta VAS difference: −0.8 ± 0.2, *p* = 0.042, Cohen’s d = 0.64) (Table 2) (Figure 4).

**Figure 5:**
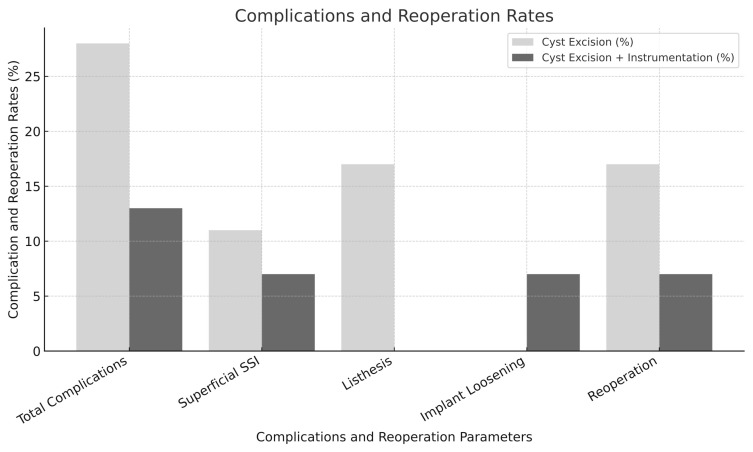
Postoperative complication and reoperation rates by surgical technique. SSI: surgical site infection.

Postoperative complications were documented in seven patients (21.2%). Specifically, the excision group experienced five complications (27.8%): two superficial surgical site infections (SSI) and three cases of progressive spondylolisthesis. The instrumentation group had two complications (13.3%): one superficial SSI and one implant loosening requiring revision. The development of postoperative spondylolisthesis was significantly higher in the excision group (16.7% vs. 0%, *p* = 0.028) (Table 3) (Figure 5).

At mean follow-up of 28 months, reoperation was performed in four patients (12.1%): three in the excision group (16.7%) and one in the instrumentation group (6.7%). Cyst recurrence occurred in three patients (9.1%): two in the excision group (11.1%) and one in the instrumentation group (6.7%). The following patients were reoperated on: in the excision group, three patients who subsequently developed listhesis and in the instrumentation group, one patient with a recurrent cyst with implant loosening. In the excision group, two of the three patients with listhesis who were reoperated on underwent recurrent cyst excision and unilateral PEEK rod stabilization, while one patient underwent unilateral PEEK rod stabilization alone. The patient who was operated on in the instrumentation group underwent recurrent cyst excision following implant revision surgery. Operation time was significantly longer in the instrumentation group (124.6 ± 23.8 vs. 82.4 ± 17.8 min, *p* < 0.001), as was hospital stay (4.0 ± 1.2 vs. 3.1 ± 1.0 days, *p* = 0.006) (Table 3).

Effect size analysis revealed that the surgical technique had the greatest impact on operative parameters (Cohen’s d = 2.02 for operation time, d = 0.82 for hospital stay) and a moderate impact on back pain improvement (d = 0.64), while showing small effects on complications (d = 0.35) and reoperations (d = 0.30) (Table 4).

The effect size analysis demonstrates that an increased operation time and hospital stay in the instrumentation group are directly associated with the complexity of the surgical technique, as opposed to the clinical outcomes. The moderate effect size for back pain improvement (Cohen’s d = 0.64) represents clinically meaningful pain reduction independent of these surgery-related factors. The instrumentation group’s superior pain control was achieved despite longer procedural time, indicating that the benefit derives from biomechanical stabilization rather than surgical duration.

## 4. Discussion

The selection of the surgical approach for lumbar synovial cysts remains contentious. In this study, we compared clinical and radiological outcomes of two groups of patients with synovial cysts at lumbar level L4–L5, one undergoing only cyst excision and the other undergoing cyst excision with unilateral instrumentation. Our research shows that both methods were effective but that determining which method to select depends on particular patient features and imaging results.

Our study demographics align with findings in the literature. The mean age of 50.8 ± 9.6 years in the excision group and 47.9 ± 10.2 years in the instrumentation group supports the finding that lumbar synovial cysts are generally more common in middle-aged populations [14]. Female predominance (61.1% in the excision group and 60.0% in the instrumentation group) likely relates to the higher incidence of degenerative processes in women, as frequently reported [11]. The female predominance reflects complex biological and social factors. The changes in estrogen levels during menopause lead to faster disk degeneration and thicker ligamentum flavum, which increases the risk of synovial cyst formation [1,11,15]. Spinal biomechanics are affected by the different paraspinal muscle activation patterns and higher prevalence of osteoporosis in women. [11] Sociocultural factors, including occupational choices, childcare-related postural stress, and delayed healthcare seeking may contribute to advanced presentation [15,16]. These gender-specific considerations should inform preoperative counseling and postoperative rehabilitation protocols. The L4–L5 level is most commonly affected due to its higher mobility and susceptibility to degenerative changes [17]. We evaluated only this level to eliminate factors complicating comparison of biomechanical properties and surgical results at different levels, allowing assessment of surgical techniques in a more homogenous group.

Both groups showed significant clinical improvement after surgery. The interpretation of our results requires evaluation of minimal clinically important differences (MCID). The established MCID for VAS pain scores ranges from 1.2 to 2.0 points while ODI requires a change of 10–15 percentage points to be considered significant. The VAS scores showed significant pain reduction in both excision and instrumentation groups with 5.0 ± 1.2 points and 5.8 ± 1.3 points, respectively, which exceed the established MCID thresholds. The ODI improvements of 42.2 ± 11.2% (excision) and 41.0 ± 11.8% (instrumentation) exceed the MCID threshold which confirmed significant functional improvement in both groups. The instrumentation group achieved better low back pain control than the excision group (Cohen’s d = 0.64, *p* = 0.042). This moderate effect size has important clinical implications. The NNT analysis shows that for every five patients treated with instrumentation instead of excision alone, one additional patient will achieve clinically meaningful improvement in back pain (>30% reduction). This finding should be weighed against increased surgical complexity and costs. These results align with similar studies regarding pain control and functional improvement after surgical treatment at the L4–L5 level [16,18]. Kim et al. reported mean VAS lumbar scores decreasing from 4.7 to 1.8 and ODI scores from 27.2% to 14.6% [18]. Tan et al. [16] reported VAS leg scores decreasing from 6.1 to 0.5 and ODI scores improving from 74.5% to 14.7% after cyst excision, similar to our findings. Gündoğdu et al. reported comparable improvements at the same level [9]. Tan et al. similarly emphasized better low back pain control in the instrumentation group [16]. These findings indicate that while both approaches effectively treat L4–L5 level synovial cysts, instrumentation may offer superior low back pain management. Although the retrospective design of our study prevents us from assessing patient satisfaction and return-to-work rates and quality-of-life measures, the superior pain control obtained from PEEK rod instrumentation may lead to improved functional capacity and an expedited return to work. The improved biomechanical stability provided by PEEK rods may lead to faster rehabilitation and improved workplace efficiency, especially for patients engaged in physically demanding tasks. Improvements in objective clinical measures (VAS, ODI) achieved by PEEK rod instrumentation suggest potential advantages for patient satisfaction and functional recovery that should be further investigated through prospective studies.

Compared to the more static stabilization systems, dynamic instrumentation offers great benefits with regard to treatment of lumbar synovial cysts. The research examined PEEK rod systems exclusively; however, given the broader context, it is necessary to consider other dynamic stabilization methods. The study does not provide a direct comparison between PEEK rods and other dynamic systems including Coflex interspinous devices and Dynesys posterior dynamic stabilization systems because significant biomechanical differences exist between them. Coflex devices provide motion preservation through interspinous distraction but may not address facet joint instability as effectively as posterior instrumentation. Dynesys systems offer posterior dynamic stabilization, however they utilize different material properties and biomechanical principles compared to PEEK rods. Future comparative studies need to compare these systems directly to determine the best treatment approaches for various patient groups and different instability patterns. The biomechanical advantages of PEEK rod instrumentation, including unilateral application, deserve emphasis. With an elastic modulus of 3–4 GPa, closer to cortical bone (18 GPa) than titanium (110 GPa), PEEK rods reduce stress shielding and the risk of degeneration of adjacent segments [4,5,6]. Our zero listhesis rate in the instrumentation group versus 16.7% in excision group supports this theoretical advantage. Dynamic systems offer flexibility which creates a more physiological biomechanical environment at adjacent segments and decreases the load. Research suggests that dynamic devices such as PEEK rods are able to provide sufficient stability while improving comfort and preserving adjacent segment integrity without the excessive flexibility of titanium alloy rods [6]. These assertions have also been confirmed through finite element analyses, where it has been observed that dynamic stabilization decreases intradiscal pressure and excessive motion of the immediate segments, thereby improving the overall outcome of the surgery [5].

Evidence indicates that excision surgery at the L4–L5 level may increase the risk oflisthesis. Siu and Stoodley found that new listhesis development after surgery occurred most frequently at the L4–L5 level [19], likely due to the biomechanical properties of this level and spinal architecture alterations during surgery. Morishita et al. noted increased listhesis risk after L4–L5 excision, particularly in older patients, and attributed this to greater mechanical loading [2]. Conversely, instrumentation surgery presents different complications. Ganga et al.’s meta-analysis focusing on the L4–L5 level reported that while instrumentation was associated with lower listhesis risk, implant-related problems remained among the potential complications [8]. According to our findings, the risk of listhesis was eliminated in the unilateral dynamic instrumentation group, and implant problems occurred at a lower rate.

Reoperation rates and recurrence risk factors significantly impact long-term outcomes in L4–L5-level surgeries. In our study, reoperation rates were 16.7% in the excision group versus 6.7% in the instrumentation group. The literature consistently reports higher reoperation rates in patients undergoing decompression alone at this level. Hellinger and Lewandrowski [20] demonstrated that level-specific biomechanical changes may contribute to higher recurrence rates in the excision group [13,20]. Page et al. found that surgeries not addressing segmental instability increase recurrence and reoperation rates, with 12.3% of decompression patients requiring reoperation [21]. Our recurrence rates were 11.1% in the excision group compared to 6.7% in the instrumentation group, suggesting that instrumentation reduces recurrence risk by enhancing segmental stability. Epstein and Agulnick similarly emphasized that non-instrumented surgery may lead to higher recurrence rates at the L4–L5 level, ref. [22] noting that instrumentation minimizes recurrence by controlling segmental mobility.

Surgical parameters at the L4–L5 level were evaluated for cost-effectiveness, particularly regarding operation time and hospital stay. We found that operation time differed by approximately 42.2 ± 5.4 min between groups (excision: 82.4 ± 17.8 min, instrumentation: 124.6 ± 23.8 min, *p* < 0.001), and hospital stay differed by 0.9 ± 0.2 days (excision: 3.1 ± 1.0 days, instrumentation: 4.0 ± 1.2 days, *p* = 0.006). Chen et al.’s meta-analysis [13] of L4–L5 synovial cysts reported a mean operation time of 85.6 ± 16.4 min for excision-only cases, similar to our excision group (82.4 ± 17.8 min). Ganga et al. [8] reported mean stays of 3.2 ± 1.1 days for excision and 4.1 ± 1.3 days for instrumentation groups in L4–L5 synovial cyst surgeries, closely matching our data. Lalanne et al. [23] similarly found that operation time increased by approximately 40 min with instrumentation but considered this acceptable given the improved postoperative stability.

The clinical decision-making process for treating L4–L5 synovial cysts needs to follow an algorithm that considers individual patient characteristics. Our study found that unilateral PEEK rod dynamic stabilization prevents listhesis and provides more effective low back pain control with the additional stability provided by instrumentation. Therefore, it can be considered that young patients performing heavy work activities and patients with instability indicators on preoperative dynamic radiographs may potentially benefit from this surgical approach, although it requires longer surgical procedures. In this case, it can be concluded that excision alone is a better option for individuals with low physical activity, the elderly, and those with medical conditions that limit the duration of surgery and demonstrate no evidence of instability on preoperative dynamic radiographs. The treatment of ambiguous cases requires shared decision-making between patients and surgeons and institutional resource utilization to achieve optimal results.

This study has multiple crucial limitations that need to be disclosed to the readers. Current study design as a retrospective analysis prevents us from obtaining complete patient-reported outcome measures which involved satisfaction scores, return-to-work durations and quality of life assessments. The study limitations are attributable to the nature of retrospective analyses rather than to problems inherent in the study design. The collection of these essential patient-centered outcomes requires prospective data collection methods. The study design as a single-center retrospective analysis produces potential selection bias because surgeons tend to choose healthier patients for excision alone, which could result in underestimating the differences between surgical complication rates. The procedures were conducted by three surgeons who might have employed different surgical methods and instrumentation standards, yet no reliability assessment between surgeons was conducted. Notwithstanding the selection of surgical approach which was based on established clinical and radiological criteria instead of surgeon preferences, retrospective nature of this study creates potential selection bias because of the fact that treatment choices were not controlled. Although we accept that the sample size for this rare pathology is small but consistent with the literature, our a priori power analysis established 80% power, and we collected more patients than needed (33 vs. 26 patients) although the post hoc analysis for secondary outcomes showed only 62% power, which might indicate a Type II error for reoperation rates while maintaining sufficient power for primary outcomes (VAS scores). The 28-month average follow-up duration does not provide sufficient time to detect long-term complications of fusion, such as adjacent segment disease and pseudarthrosis, and late implant failure, which usually appear between 5 and 10 years post-surgery. The absence of standing MRI or CT myelography might have resulted in an underestimation of spinal stenosis and dynamic instability and the lack of patient satisfaction data, return-to-work information, and quality-of life-assessments prevent complete outcome evaluation. This study’s single-center design and single-level focus limit the ability to generalize findings to other patient groups and spinal regions.

However, the strengths of our study include a more homogeneous patient group by comparing cases at the same spinal level, follow-up of patients with detailed clinical and radiological data, and standardization of surgical techniques. For future studies, multicenter and prospective randomized studies with larger patient groups are needed for the treatment of synovial cysts at the L4–L5 level. In addition, studies evaluating long-term results specific to this level and including cost-effectiveness analyses should be planned. The combination of upright MRI with finite element modeling in advanced imaging protocols would improve instability risk prediction. Research on biomarkers for cyst recurrence and machine learning algorithms for surgical selection shows great promise.

## 5. Conclusions

In conclusion, in the surgical treatment of lumbar synovial cysts at the L4–L5 level, both cyst excision alone and the excision technique with unilateral instrumentation are better in terms of VAS score treatment options. Both approaches provided satisfactory results in terms of pain control and functional improvement. However, it was observed that the instrumentation group had better control of low back pain and lower risk of listhesis. In contrast, the operation time and hospital stay were shorter in the excision-only group.

Clinical Decision-Making Algorithm:

Our research leads to the following evidence-based decision framework for the treatment of L4–L5 synovial cysts:

- The assessment process should evaluate with together patient age, activity level, dynamic instability on flexion-extension radiograph and comorbid conditions.

The treatment choice depends on the following factors:

- The recommended treatment for patients under 65 years old who exhibit high levels of physical activity and have grade 1 dynamic instability or occupational requirements for spinal stability involves cyst excision and unilateral PEEK rod instrumentation. For patients above 65 years old who exhibit low activity levels, having significant comorbidities limiting surgical duration and no dynamic instability, cyst excision alone is recommended.
- The analysis should compare the additional 42 min surgical time and 0.9-day hospital stay extension with the superior pain management and listhesis prevention outcomes.

This study provides evidence-based recommendations for choosing surgical approaches when treating L4–L5 synovial cysts. The unilateral instrumentation technique provides better control of back pain and prevention of listhesis but requires more complex surgery and higher initial costs. The decision algorithm should integrate patient age, activity level, radiological findings, and economic factors. Younger active patients with instability risk factors should receive instrumentation as the optimal treatment despite its higher initial expenses. Patients who are elderly or have low activity levels can achieve satisfactory results through excision alone. In the light of these findings, we can say that the choice of techniques in the surgical treatment of synovial cysts at the L4–L5 level should be made by considering patient-specific factors. Particularly in patients with a high risk of instability, the addition of instrumentation may be considered.

## Figures and Tables

**Table 1:** Demographic and clinical characteristics.

Demographic and Clinical Characteristics	Cyst Excision (*n* = 18)	Cyst Excision + Instrumentation (*n* = 15)	*p*-Value
Age (years) *	50.8 ± 9.6	47.9 ± 10.2	0.412 †
Gender ‡			0.892 §
- Female	11 (61.1%)	9 (60.0%)	
- Male	7 (38.9%)	6 (40.0%)	
Symptom duration (months) *	8.1 ± 4.0	7.6 ± 3.8	0.528 †
Presenting symptoms ‡			
- Back pain	16 (88.9%)	13 (86.7%)	0.845 §
- Leg pain	17 (94.4%)	14 (93.3%)	0.898 §
- Neurogenic claudication	11 (61.1%)	9 (60.0%)	0.932 §
Comorbid pathologies ‡			
- Spondylolisthesis	5 (27.8%)	4 (26.7%)	0.812 §
- Spinal stenosis	8 (44.4%)	7 (46.7%)	0.924 §

Notes: Values are presented as mean ± standard deviation. The asterisk (*) denotes continuous variables. *p*-values were calculated using the independent samples *t*-test † or chi-square test/Fisher’s exact test §. Data are presented as numbers and percentages ‡.

**Table 2:** Clinical outcomes and comparison.

Clinical Parameters	Cyst Excision (*n* = 18)	Cyst Excision + Instrumentation (*n* = 15)	*p*-Value †
VAS back pain *			
- Preoperative	7.3 ± 1.3	7.9 ± 1.1	0.068
- Postoperative	2.3 ± 1.0	2.1 ± 1.2	0.078
- Delta VAS back pain	5.0 ± 1.2	5.8 ± 1.3	0.042
- *p*-value ‡	<0.001	<0.001	
VAS leg pain *			
- Preoperative	7.5 ± 1.2	7.3 ± 1.3	0.632
- Postoperative	2.0 ± 1.1	2.2 ± 1.0	0.558
- Delta VAS leg pain	5.5 ± 1.3	5.1 ± 1.2	0.362
- *p*-value ‡	<0.001	<0.001	
ODI (%) *			
- Preoperative	63.8 ± 11.8	61.9 ± 12.6	0.502
- Postoperative	21.6 ± 8.2	20.9 ± 8.8	0.812
- Delta ODI	42.2 ± 11.2	41.0 ± 11.8	0.608
- *p*-value ‡	<0.001	<0.001	

Values are presented as mean ± standard deviation. The asterisk (*) denotes continuous variables. *p*-values for between-group comparisons were calculated using the independent samples *t*-test †, while within-group comparisons were analyzed using the paired *t*-test ‡.

**Table 3:** Complications and follow-up parameters.

Complications and Follow-Up Parameters	Cyst Excision (*n* = 18)	Cyst Excision + Instrumentation (*n* = 15)	*p*-Value
Complications ‡			
- Total	5 (27.8%)	2 (13.3%)	0.208 §
- Superficial SSI	2 (11.1%)	1 (6.7%)	0.512 §
- Listhesis	3 (16.7%)	0 (0%)	0.028 §
- Implant loosening	0 (0%)	1 (6.7%)	0.102 §
Follow-up parameters			
- Follow-up duration (months) *	28.2 ± 4.0	27.6 ± 4.4	0.612 †
- Reoperation ‡	3 (16.7%)	1 (6.7%)	0.318 §
- Recurrence ‡	2 (11.1%)	1 (6.7%)	0.382 §
- Operation time (minutes) *	82.4 ± 17.8	124.6 ± 23.8	<0.001 †
- Hospital stay (days) *	3.1 ± 1.0	4.0 ± 1.2	0.006 †

Values are presented as mean ± standard deviation. The asterisk (*) denotes continuous variables. *p*-values for between-group comparisons were calculated using the independent samples *t*-test † or chi-square test/Fisher’s exact test §. Data are presented as numbers and percentages ‡.

**Table 4:** Intergroup comparison and effect size analysis.

Clinical Parameters	Difference (Group 1, Group 2)	*p*-Value †	Cohen’s d
Demographic data			
- Age	2.9 ± 1.1	0.412	0.29
- Symptom duration	0.5 ± 0.3	0.528	0.13
VAS scores			
- Delta VAS back pain	−0.8 ± 0.2	0.042	0.64
- Delta VAS leg pain	0.4 ± 0.2	0.362	0.31
Functional outcomes			
- Delta ODI	1.2 ± 0.7	0.608	0.10
Surgical parameters			
- Operationtime	−42.2 ± 5.4	<0.001	2.02
- Hospital stay	−0.9 ± 0.2	0.006	0.82
Complications			
- Total complication rate	14.5%	0.208	0.35
- Reoperation rate	10.0%	0.318	0.30

Values are presented as absolute mean differences ± standard error for continuous variables or absolute percentage differences for categorical variables. Group 1 = cyst excision only; Group 2 = cyst excision + instrumentation group. Negative values are indicative of lower values in Group 1, while positive values are indicative of higher values in Group 1. Standard error represents standard error of mean difference. Statistical significance was considered at *p* < 0.05 †. Cohen’s d indicates effect size, where 0.2 represents small effect, 0.5 medium effect, and 0.8 large effect.

## Data Availability

The data presented in this study are available on request from the corresponding author due to privacy and ethical restrictions.
